# Recent advancements in image‐enhanced endoscopy in the pancreatobiliary field

**DOI:** 10.1002/deo2.382

**Published:** 2024-05-14

**Authors:** Haruka Toyonaga, Tsuyoshi Hayashi, Kazuki Hama, Ryo Ando, Tatsuya Ishii, Kenta Yoshida, Toshifumi Kin, Masayo Motoya, Kuniyuki Takahashi, Akio Katanuma

**Affiliations:** ^1^ Center for Gastroenterology Teine Keijinkai Hospital Hokkaido Japan

**Keywords:** endoscopy, image‐enhanced endoscopy, pancreatobiliary, red dichromatic imaging, texture and color enhancement imaging

## Abstract

Image‐enhanced endoscopy (IEE) has advanced gastrointestinal disease diagnosis and treatment. Traditional white‐light imaging has limitations in detecting all gastrointestinal diseases, prompting the development of IEE. In this review, we explore the utility of IEE, including texture and color enhancement imaging and red dichromatic imaging, in pancreatobiliary (PB) diseases. IEE includes methods such as chromoendoscopy, optical‐digital, and digital methods. Chromoendoscopy, using dyes such as indigo carmine, aids in delineating lesions and structures, including pancreato‐/cholangio‐jejunal anastomoses. Optical‐digital methods such as narrow‐band imaging enhance mucosal details and vessel patterns, aiding in ampullary tumor evaluation and peroral cholangioscopy. Moreover, red dichromatic imaging with its specific color allocation, improves the visibility of thick blood vessels in deeper tissues and enhances bleeding points with different colors and see‐through effects, proving beneficial in managing bleeding complications post‐endoscopic sphincterotomy. Color enhancement imaging, a novel digital method, enhances tissue texture, brightness, and color, improving visualization of PB structures, such as PB orifices, anastomotic sites, ampullary tumors, and intraductal PB lesions. Advancements in IEE hold substantial potential in improving the accuracy of PB disease diagnosis and treatment. These innovative techniques offer advantages paving the way for enhanced clinical management of PB diseases. Further research is warranted to establish their standard clinical utility and explore new frontiers in PB disease management.

## INTRODUCTION

The use of gastrointestinal endoscopes began in the 19th century with the “light conductor,” consisting of a tube and mirrored mechanism for visual inspection of the human body.^1^ Advancements in fiber‐optic technology in the 1950s and the introduction of electronic videoendoscopy in the 1980s improved image clarity and flexibility.^2^ Electronic videoendoscopy converts electronic signals into images using semiconductor elements and allows various forms of electronic image processing and analysis.

Endoscopy is useful for diagnosing gastrointestinal (GI) diseases; however, all GI diseases cannot be detected using conventional white‐light imaging (WLI). Consequently, image‐enhanced endoscopy (IEE) has been developed to improve diagnostic capabilities by enhancing endoscopic images using dyes, special light sources, and image processing.^3^

Endoscopic treatment of pancreatobiliary (PB) diseases has been developed on the basis of the devices and technologies used in the GI field. IEE, which is widely used in the GI field, has also recently been applied in the PB field. New types of IEE, such as texture and color enhancement imaging (TXI) and red dichromatic imaging (RDI), featured in Olympus's latest endoscopic system, EVIS X1 (Olympus Co.), were launched in Europe in April 2020 and in Japan in July 2020, and have garnered increasing attention (Table 1).

**TABLE 1:** Summary of the current literature about image‐enhanced endoscopy: texture and color enhancement imaging, and red dichromatic imaging.

Type of image‐enhanced endoscopy	Clinical applications	Author, year of publication^ref.^	Study design	Result
**RDI**	**Detection of bleeding points**	Inoue et al., 2022^24^	Case report	During hemostasis of post‐EST bleeding, endoscopic visibility improved; bleeding points were identified, ongoing bleeding or cessation was determined, and mental stress was reduced (RDI Modes 1 and 2).
		Nakamura et al., 2022^25^		
		Toyonaga et al., 2023^26^, ^27^		
	**POCS**	Kimura et al., 2023^28^	Case report	The bleeding point of the intraductal biliary hemorrhage was identified during POCS (RDI Mode 2).
		Koiwai et al., 2023^29^	Case report	POCS showed the biliary lesions and identified the lateral margin (RDI Mode 3).
		Tanisaka et al., 2023^30^		
		Matsumoto et al., 2023^31^		
**TXI**	**Observation of pancreatobiliary orifices**	Miyaguchi et al., 2023^34^	Retrospective cohort study	TXI was useful for recognizing the bile duct opening pattern and the separate and septal types of the duodenal papilla (TXI Mode 1).
		Tanisaka et al., 2024^35^	Retrospective cohort study	The biliary cannulation time was shortened in patients who underwent Roux‐en‐Y gastrectomy via single‐balloon enteroscopy‐assisted ERCP (TXI Mode1).
		Toyonaga et al., 2022^36^	Case report	Visualization of the biliary orifice on an interdiverticular papilla or incision surface improved after precut papillotomy (TXI Modes 1 and 2).
		Tanisaka et al., 2024^37^		
		Toyonaga et al., 2024^38^	Retrospective cohort study	Visualization of the biliary orifice on the incision surface was improved after needle knife precut papillotomy. The accuracy rate, visibility, and color difference between the biliary orifice and surrounding tissue were improved (TXI Mode 2).
	**Ampullary tumors**	Tanisaka et al., 2023^39^	Case report	Visualization of ampullary tumor margins and pancreatobiliary orifices was improved after endoscopic papillectomy (TXI Modes 1 and 2).
		Toyonaga et al., 2023^40^		
	**Detection of cholangio‐/pancreato‐jejunal anastomosis**	Toyonaga et al., 2022^41^	Case report	Visualization of pancreato‐/cholangio‐jejunal anastomotic stenoses, which were difficult to identify, was improved (TXI Modes 1 and 2).
		Tanisaka et al., 2023^42^		
		Takenaka et al. 2023^43^		
	**POCS**	Tanisaka et al., 2023^30^	Case report	Visualization of biliary lesions was improved during POCS (TXI).

Abbreviations: ERCP, endoscopic retrograde cholangiopancreatography; EST, endoscopic sphincterotomy; POCS, peroral cholangioscopy; RDI, red dichromatic imaging; TXI, texture and color enhancement imaging.

Herein, we describe the usefulness of IEE, including TXI and RDI, in the PB field.

## MECHANISMS AND EQUIPMENT OF IEE

**FIGURE 1:**
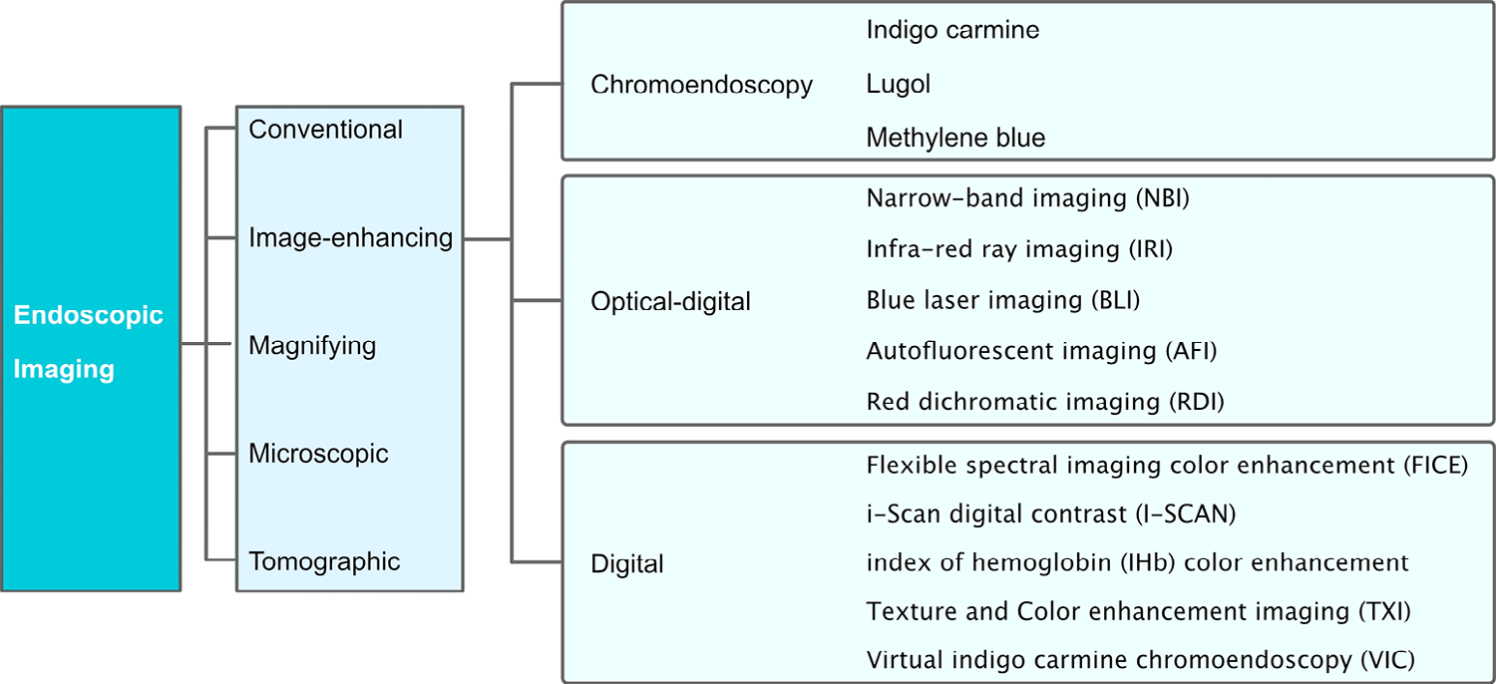
Endoscopic imaging‐object‐oriented classification.

IEE refers to a collective term for methods that facilitate the distinction between normal mucosa and lesions by enhancing the color tones and boundaries.^4^ This is achieved through the dispersion of liquids such as dyes or stains (chromoendoscopy method), altering the special light source (optical‐digital method), or applying digital processing (digital method; Figure 1).

### Chromoendoscopy method

Chromoendoscopy is an early form of IEE that involves the application of dyes or stains (e.g., indigo carmine, Lugol, and methylene blue) directly onto the mucosal surface or lesions, which clarify the boundaries with the surrounding structures or stains the tissues to facilitate observation. Indigo carmine is frequently used for PB diseases.

#### Indigo carmine

##### Ampullary tumors

**FIGURE 2:**
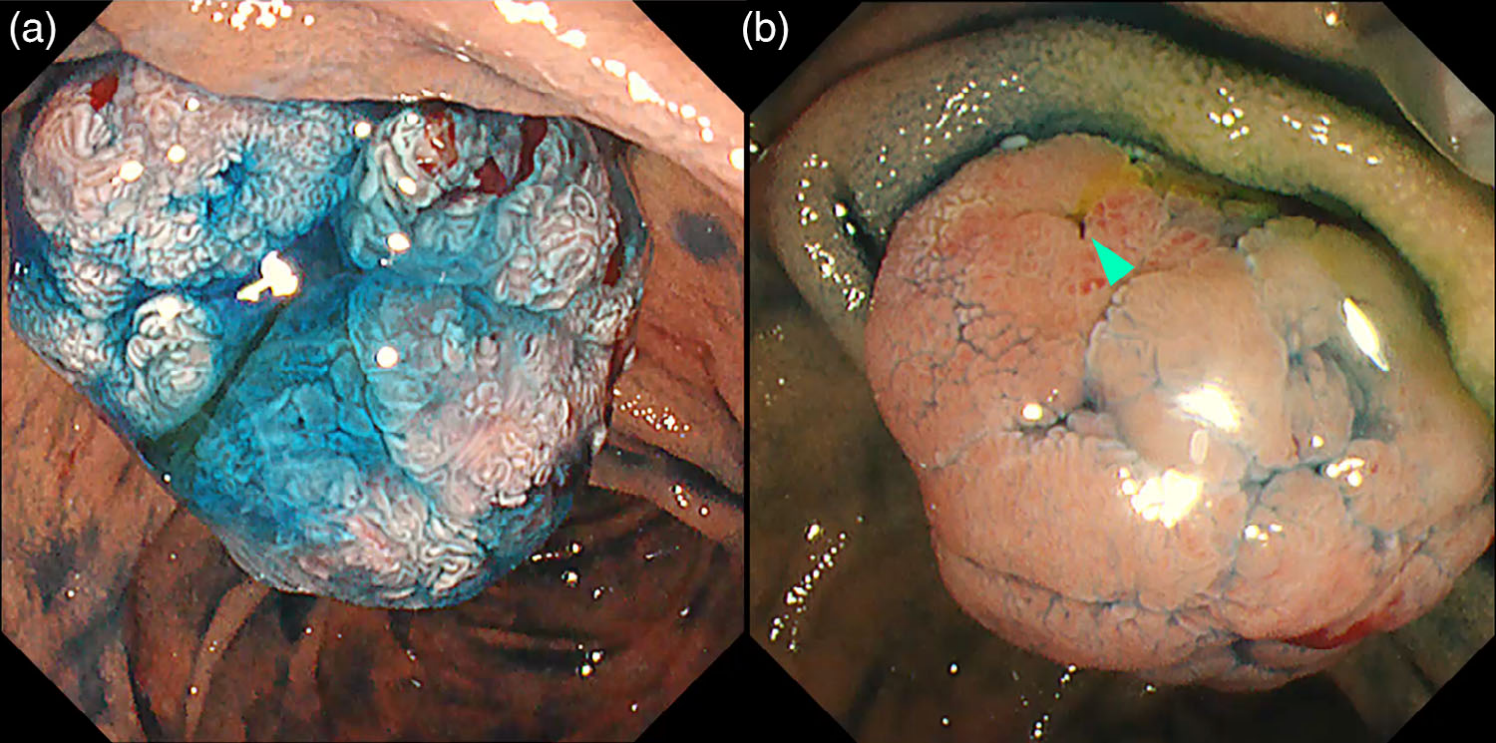
Chromoendoscopy with indigo carmine for ampullary tumors. Indigo carmine enhances the surface structure and boundaries of the tumors (a) and clarifies bile secretion to facilitate the recognition of the biliary orifice (green arrowhead) (b).

While considering the resection of ampullary tumors, it is crucial to assess the invasion depth and lateral extension into the PB ducts. Moreover, to determine the feasibility of en bloc resection, an accurate evaluation of the lateral extension toward the duodenal mucosal side is essential. Similar to the spraying of indigo carmine for GI lesions, the surface structural differences between ampullary tumors and the background mucosa enable clear recognition of the boundaries between the lesions and background mucosa.^5^, ^6^ Additionally, during treatment, the use of indigo carmine to clearly delineate the boundaries before excision can be useful for avoiding residual lesions (Figure 2a,b).

##### Detection of afferent limbs during balloon enteroscopy‐assisted endoscopic retrograde cholangiopancreatography

**FIGURE 3:**
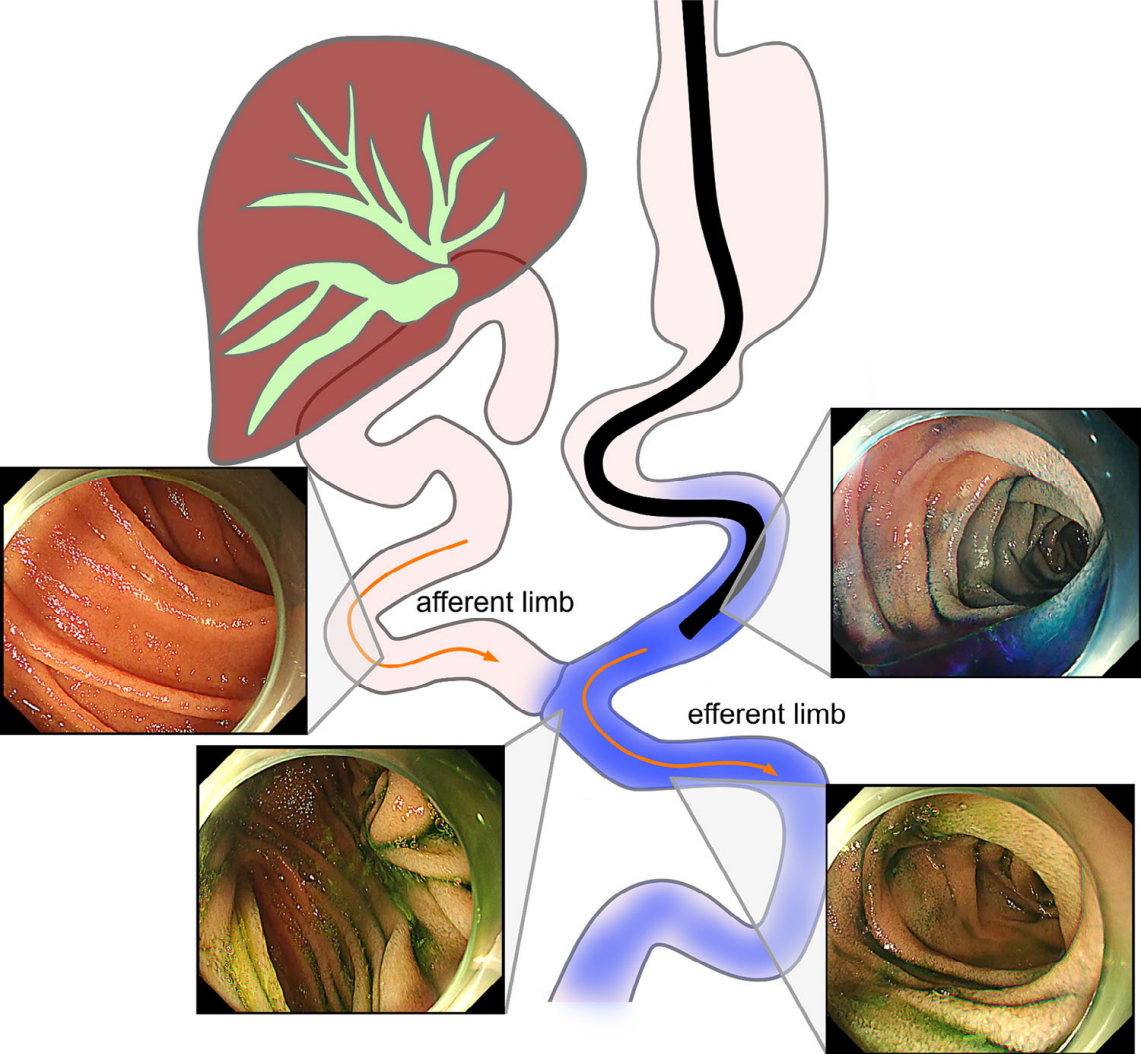
Intraluminal injection of indigo carmine for identifying the afferent limb during balloon enteroscopy‐assisted endoscopic retrograde cholangiopancreatography.

To treat PB diseases in cases of postoperative intestinal reconstruction, it is necessary to reach the papilla or anastomosis site. However, identifying the correct route to these locations, that is the afferent limb, can sometimes be challenging. Yano et al.^7^ reported that an intraluminal injection of indigo carmine facilitates the identification of the afferent limb of the Roux‐en‐Y anastomosis during balloon enteroscopy‐assisted endoscopic retrograde cholangiopancreatography (BE‐ERCP). This technique is based on the mechanism by which only a small amount of indigo carmine flows into the afferent limb, while it easily flows into the efferent limb by peristalsis (Figure 3).

##### Detection of pancreato‐/cholangio‐jejunal anastomosis

**FIGURE 4:**
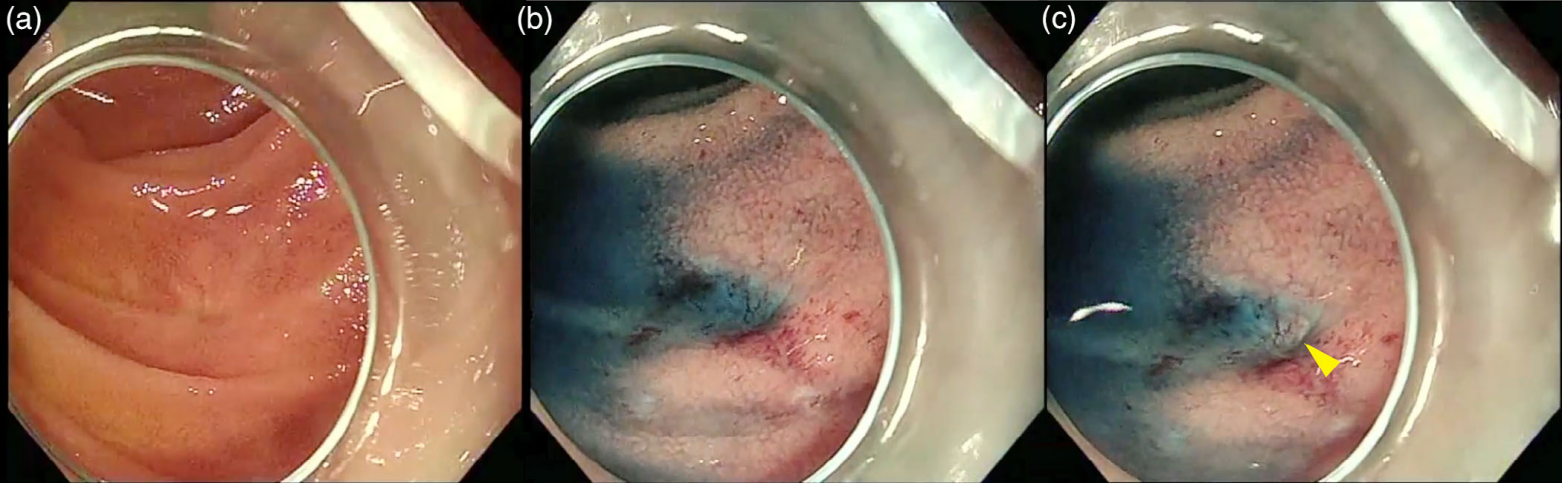
(a) In white light imaging, the pancreato‐jejunal anastomosis stenosis is not discernible. (b) Spraying indigo carmine reveals the depression of the pancreato‐jejunal anastomosis stenosis. (c) Indigo carmine accumulation in the depression is diluted by the secreted pancreatic juice (yellow arrowhead).

Pancreato‐/cholangio‐jejunal anastomotic strictures (P/CJSs) are delayed complications of hepatic PB surgery. BE‐ERCP is widely used to treat P/CJS. Reaching the anastomosis with a balloon endoscope and identifying the P/CJS are both difficult. There are several techniques for detecting P/CJS, one of which is the application of indigo carmine. Kin et al.^8^ reported that spraying indigo carmine near the P/CJS in the afferent limb covers the mucosal surface; however, on the P/CJS, it is locally diluted by bile and pancreatic juices that are secreted in small amounts. This dilution creates a difference in the color tone from the surrounding area, allowing identification of the P/CJS location (Figure 4a–c).

### Optical‐digital method

The optical‐digital method involves adapting the optical characteristics of the illuminating light or using a light source with different characteristics from ordinary white light.^4^ In addition, the signals are processed to yield enhanced images. The optical‐digital method usually encompasses narrow‐band imaging (NBI), infrared ray imaging, blue laser imaging, autofluorescent imaging, and RDI.^9^ NBI and RDI are widely used and reported in the PB field.

#### Narrow‐band imaging

The NBI endoscopic system (Olympus Medical Systems) was developed by Sano et al.^10^, ^11^ NBI illuminates the observation area with two narrow‐band wavelengths, blue (390–445 nm) and green (530–550 nm), which are most intensely absorbed by hemoglobin in the blood, enabling enhancement and display of the capillaries in the mucosal surface and fine patterns of the mucosa. Cancer is characterized by an increased cellular intake of nutrients for growth, leading to an increase in the capillaries and a change in the mucosal surface to a more complex pattern as the cancer expands. Therefore, NBI supports the early detection of cancer. In the PB field, NBI is used during peroral cholangioscopy (POCS) and examination of ampullary tumors.

##### Ampullary tumors

**FIGURE 5:**
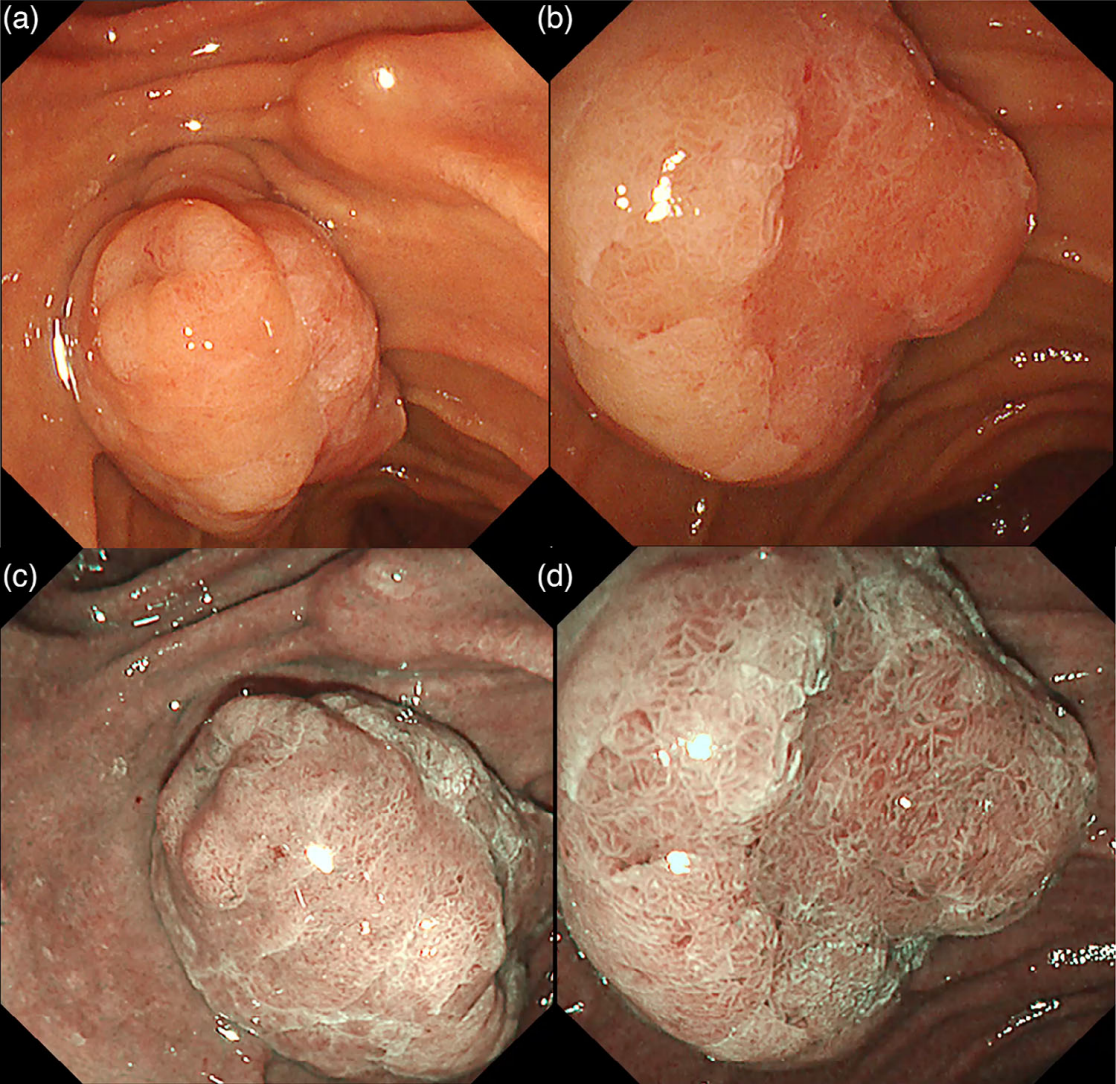
Narrow‐band imaging of ampullary tumors. With narrow‐band imaging, it is easier to recognize abnormalities in the surface and vascular structures. (a, b) White light imaging; (c, d) narrow‐band imaging.

As mentioned in the section on “Chromoendoscopy‐indigo carmine,” IEE has been reported to be useful in the evaluation of lateral extension of ampullary tumors. Several studies have shown that NBI is also useful for the close examination of ampullary tumors (Figure 5a–d).

Itoi et al.^5^ conducted a prospective cohort study involving consecutive 14 patients with ampullary tumors to assess the enhancement of the lateral margins of ampullary tumors by comparing NBI with indigo carmine. The authors reported that in all lesions, either indigo carmine or NBI was superior to conventional WLI except in one case where indigo carmine was used. The ability of NBI to emphasize the tumor margin was significantly better than that of indigo carmine (*p* = 0.05). Furthermore, NBI does not require dye spraying and repeated comparisons between WLI and NBI can be performed by pushing a button.

For discriminating between benign and malignancy lesions, Uchiyama et al.^12^ reported the usefulness of magnifying endoscopy with NBI. Magnifying endoscopy NBI findings were classified as I, oval‐shaped villi; II, pinecone/leaf‐shaped villi; or III, irregular/nonstructured. Tortuous, dilated, and network‐like vessels noted on ampullary lesions in magnifying endoscopy NBI were defined as abnormal vessels. All adenomas and adenocarcinomas had type II and/or III surface structures. Patients whose ampulla had a type I surface structure exhibited only inflammatory or hyperplastic changes. In addition, abnormal vessels were observed only in adenocarcinomas and never in adenomas.

##### Peroral cholangioscopy

POCS is useful for diagnosing indeterminate biliary tract diseases and detecting preoperative mucosal cancerous extensions in extrahepatic bile duct cancers. There are several types of POCS systems, and the capability to use NBI is available with Olympus's mother‐baby‐type POCS, CHF‐B260, and CHF‐B290.

In cholangioscopy, atypical vessels and surface structures have long been recognized as findings suggestive of malignancy and the usefulness of NBI has also been reported.^13^, ^14^, ^15^ In a recent large‐scale retrospective study involving 458 patients who underwent POCS with NBI, Shin et al.^16^ reported that papillary lesions (*p* = 0.041), nodular lesions (*p* = 0.044), and vessels that were either irregularly or regularly dilated and tortuous (*p* = 0.004 and *p* = 0.006, respectively) were associated with intraductal neoplasms of the bile duct.

Osanai et al.^17^ conducted a prospective multicenter study on the efficacy of video POCS with NBI and found that POCS with NBI could distinguish benign from malignant lesions with 92.1% accuracy in 87 patients. Itoi et al.^18^ reported that the ability to determine the margin of the intraductal papillary neoplasm of the bile duct (IPNB) of POCS using NBI is superior to that of conventional observation and may be helpful for the evaluation of fine mucosal structures, facilitating the diagnosis of tumor spread in patients with IPNB. In an ex vivo comparison study, Ishida et al.^19^ reported that a magnifying endoscope with NBI is superior to a conventional video cholangioscope with NBI for detailed visualization of the bile duct mucosa, especially in patients with minimal inflammation.

**FIGURE 6:**
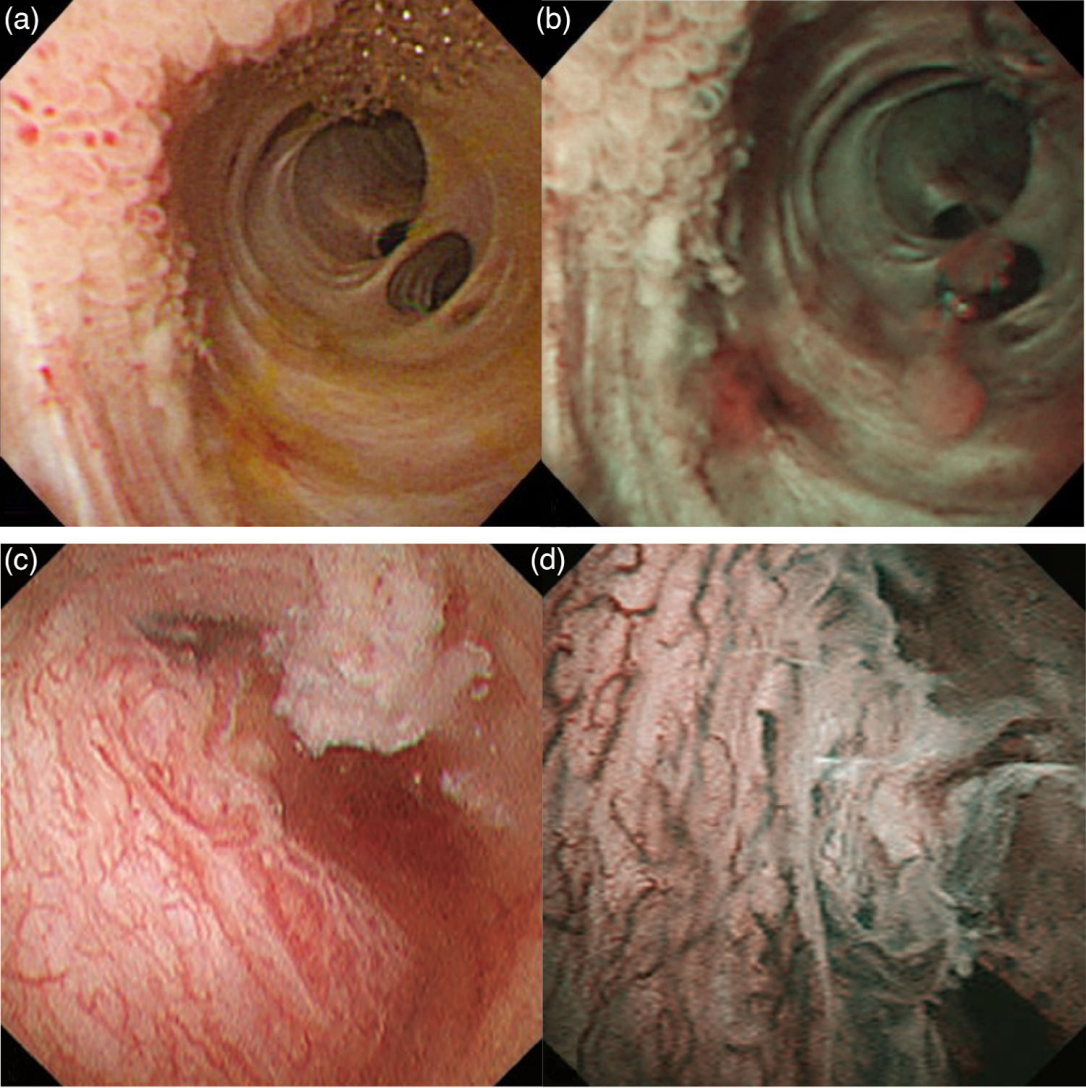
Peroral cholangioscopy with narrow‐band imaging of bile duct lesions. (a) Laterally spreading papillary biliary lesions in white light imaging and (b) in narrow‐band imaging. (c) Nodular bile duct cancer with irregularly dilated and tortuous vessels in white light imaging and (d) in narrow‐band imaging.

NBI can enhance mucosal structures and vessels, enabling the observation of minute changes in mucosal surfaces (Figure 6a–d). However, if bile or blood is present in the bile ducts during NBI, the bile appears red and blood appears brown, reducing visibility. Therefore, it is necessary to repeatedly wash and aspirate the bile duct with saline solution during the examination. To further improve visibility and diagnostic performance, a magnification function is expected to be added to cholangioscopy in the future.

#### Red dichromatic imaging

RDI has been installed in the latest endoscopy system, EVIS X1. It improves the visibility of thick blood vessels in deeper tissues and enhances bleeding points with different colors and see‐through effects. The light source of the EVIS X1 consists of five light‐emitting diodes (LEDs; violet, blue, green, amber, and red). While all five LEDs emit light during WLI observations, only three longer‐wavelength LEDs, green (520–550 nm), amber (595–610 nm), and red (620–640 nm), emit light during RDI observations.

**FIGURE 7:**
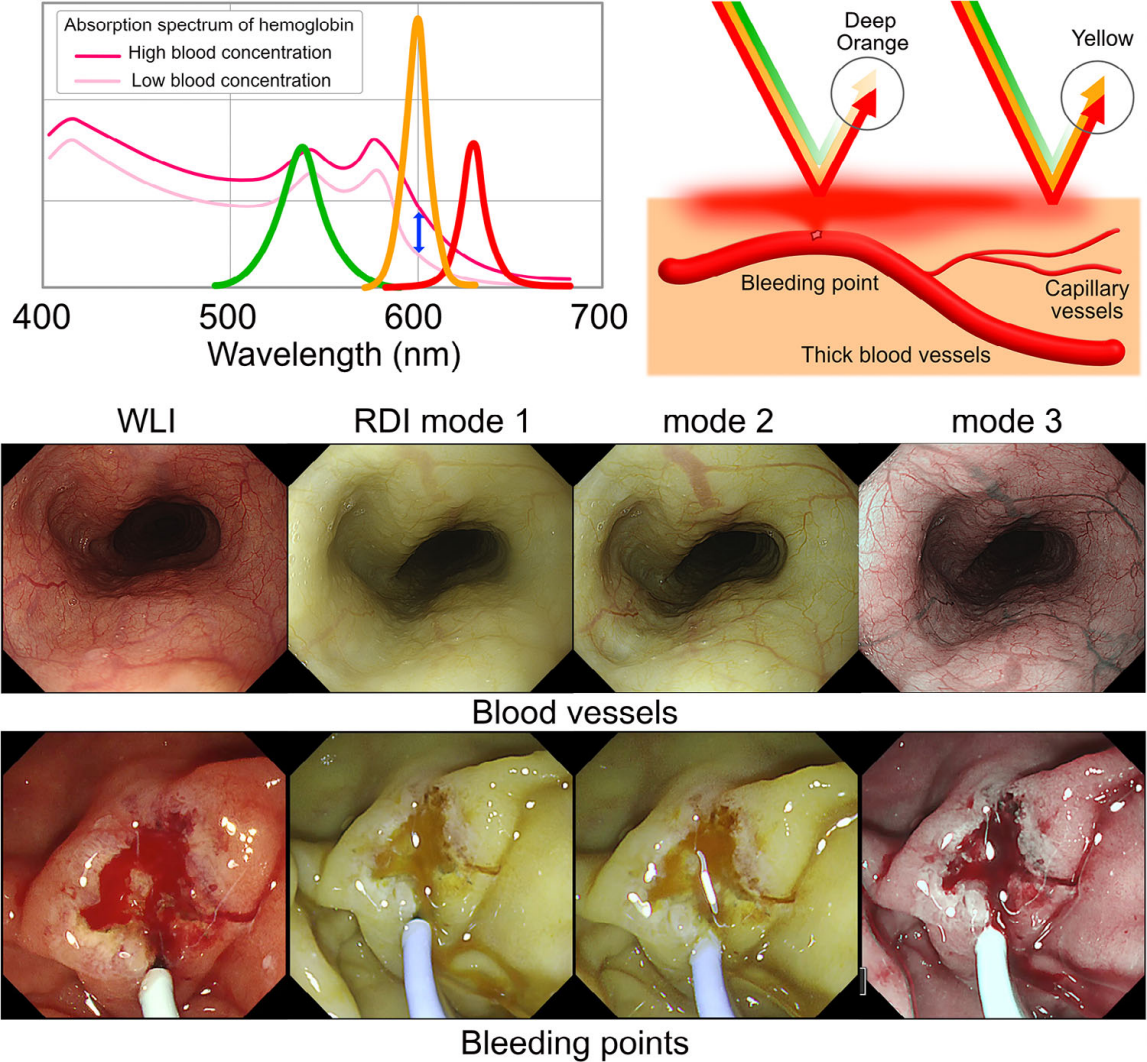
Main mechanism of red dichromatic imaging.

The main mechanism of RDI,^9^, ^20^ which enables distinct visualization of the bleeding points, relies on the difference in the hemoglobin absorption at 600 nm (amber‐colored) and 630 nm (red‐colored). Because hemoglobin strongly absorbs light at 600 nm, the reflected light is greatly attenuated at bleeding points with higher concentrations of hemoglobin than in the surrounding diluted blood. In contrast, hemoglobin weakly absorbs light at 630 nm, and the reflected light is weakly attenuated, regardless of the hemoglobin concentration (Figure 7). Thus, when observing bleeding using RDI, the bleeding points appear orange in contrast to the surrounding yellow color.

RDI has three observational modes, Mode 1, Mode 2, and Mode 3, which are determined by color allocation and enhancement. RDI Mode 1 is the basic mode, producing an overall yellow‐tone image, with deep vessels and dense blood depicted in orange. Mode 2 further emphasizes the red tones, rendering dense blood a deep orange‐red color. To recognize the bleeding points, Mode 2 offers a stronger color contrast between different blood concentrations, making them easier to identify. Meanwhile, Mode 3 depicts deep vessels in green, giving an impression similar to NBI images.

RDI Mode 3 allows observation of the mucosal tissue and blood vessels under the bile juice because the illumination lights employed by RDI are weakly absorbed by the bilirubin in bile juice, unlike in NBI.

##### Detection of bleeding points

In the GI field, numerous studies have shown that RDI is useful for the control of bleeding through improved visibility of the deep thick vessels^20^ and bleeding points in hemorrhagic ulcers^21^, ^22^ and esophageal variceal rupture.^23^ However, in the biliopancreatic area, only some case reports^24^, ^25^, ^26^ are available and no large‐scale studies have been performed.

**FIGURE 8:**
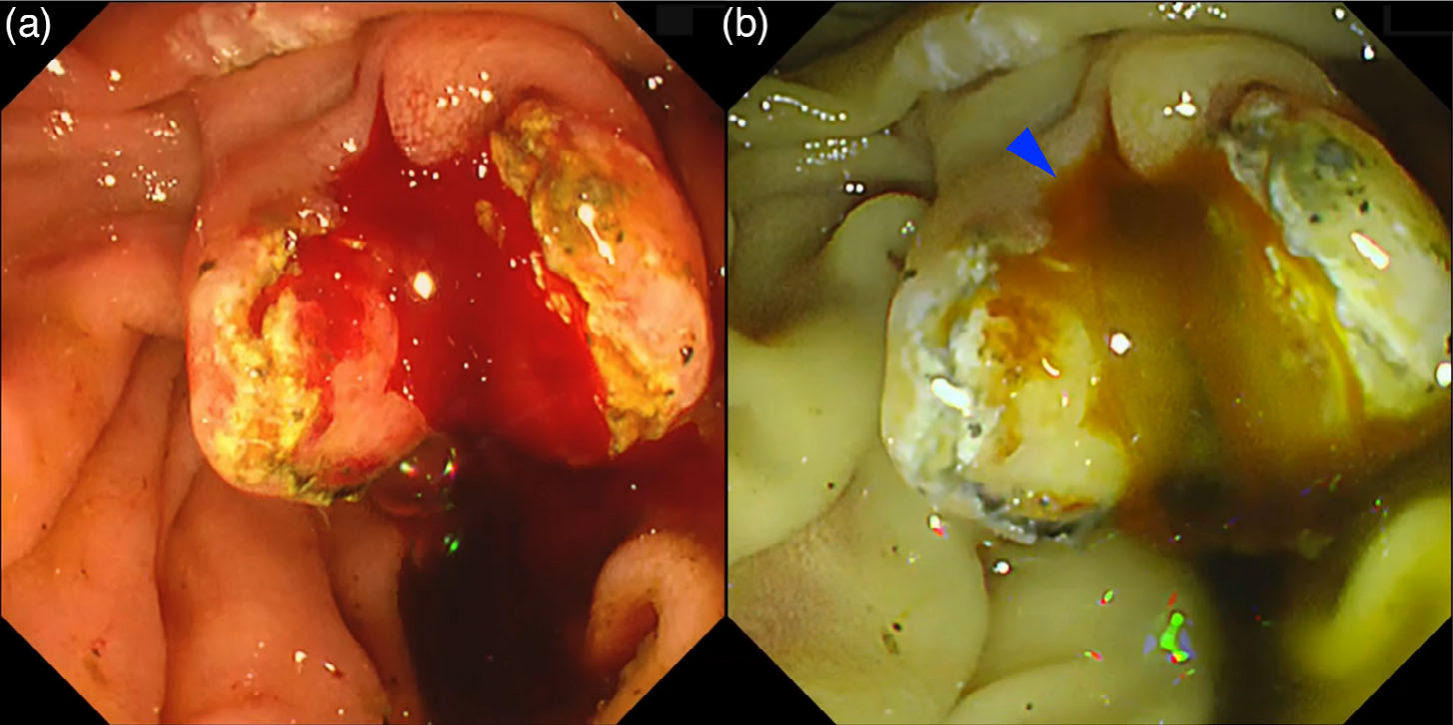
Detection of the bleeding point (blue arrowhead) with red dichromatic imaging during post‐endoscopic sphincterotomy bleeding. (a) White light imaging and (b) red dichromatic imaging Mode 2. Owing to the characteristics of red dichromatic imaging, diluted blood appears transparent and bleeding points are depicted as orange.

**FIGURE 9:**
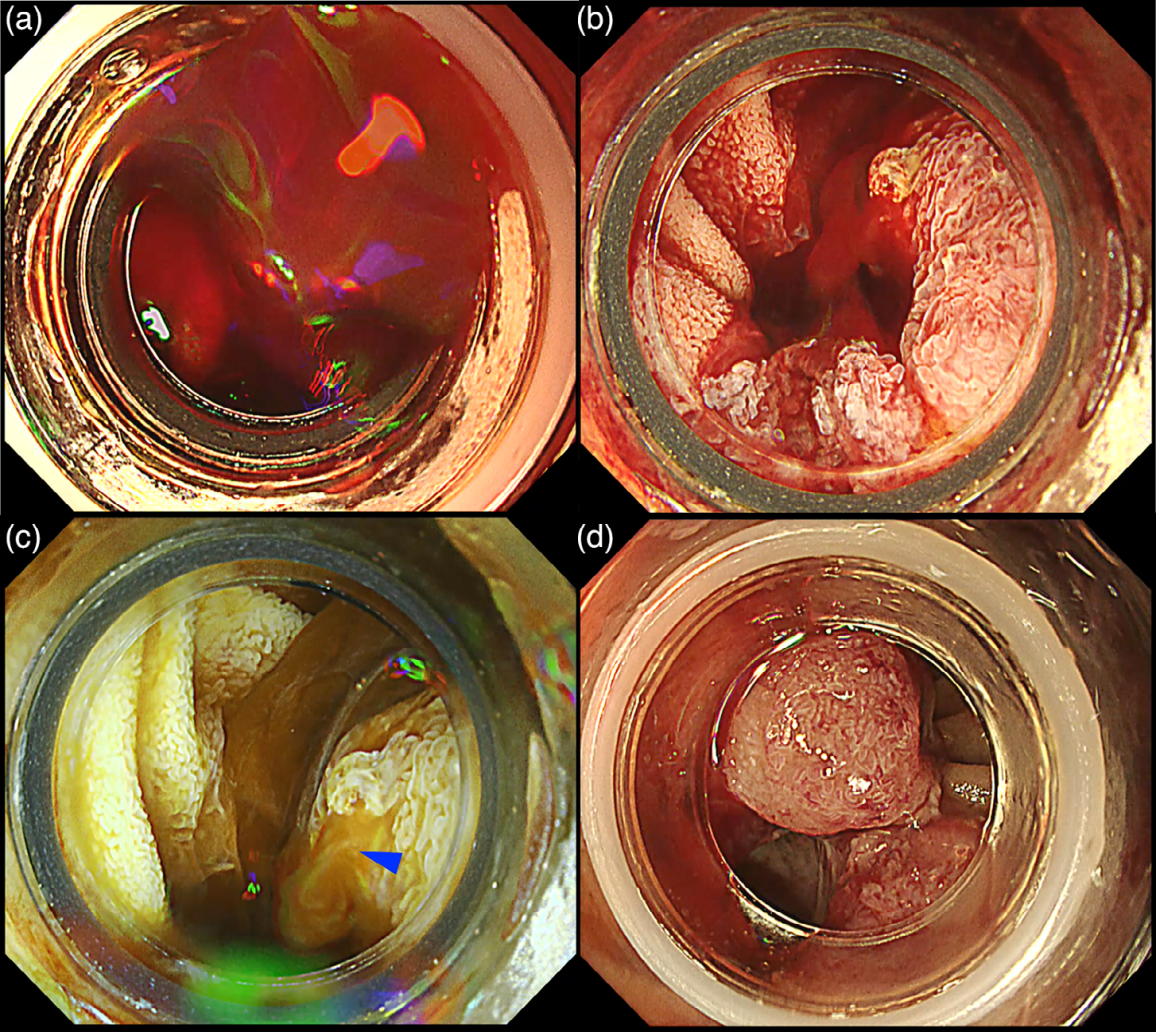
Endoscopic variceal ligation for massive variceal hemorrhage around the cholangio‐jejunal anastomotic site after pancreaticoduodenectomy. (a) Poor visibility due to massive bleeding. (b) Gel immersion technique using Viscoclear (Otsuka Pharmaceutical Factory, Inc., Tokushima, Japan) secures visibility. (c) Red dichromatic imaging further improves the visibility of bleeding points (blue arrowhead) and reduces the mental load caused by the color of the blood. (d) Successful achievement of hemostasis with endoscopic variceal ligation.

In the biliopancreatic area, bleeding is most frequently observed after endoscopic sphincterotomy (EST), and bleeding points are often hidden beneath mucosal folds, bile, or pooled blood, which can make the achievement of hemostasis challenging. In RDI, fresh and dense blood is depicted as an orange flow, allowing for the identification of bleeding points by following this trail (Figure 8a,b). In WLI, both fresh blood and diluted pooled blood have the same red color. Diluted blood appears translucent in RDI, facilitating the identification of the bleeding source and making it easier to determine whether bleeding is ongoing or hemostasis has been achieved. Additionally, the use of the gel immersion method in conjunction with RDI can further clarify the location of bleeding points^27^ (Figure 9a–d).

When switching to RDI during massive bleeding, the overall redness on the screen is subdued and depicted in yellow to orange shades. This reduces the psychological stress on the operator and facilitates a calm approach to the procedure. Careful observation and identification of the bleeding point with good visibility can contribute to appropriate hemostasis.

##### Peroral cholangioscopy

**FIGURE 10:**
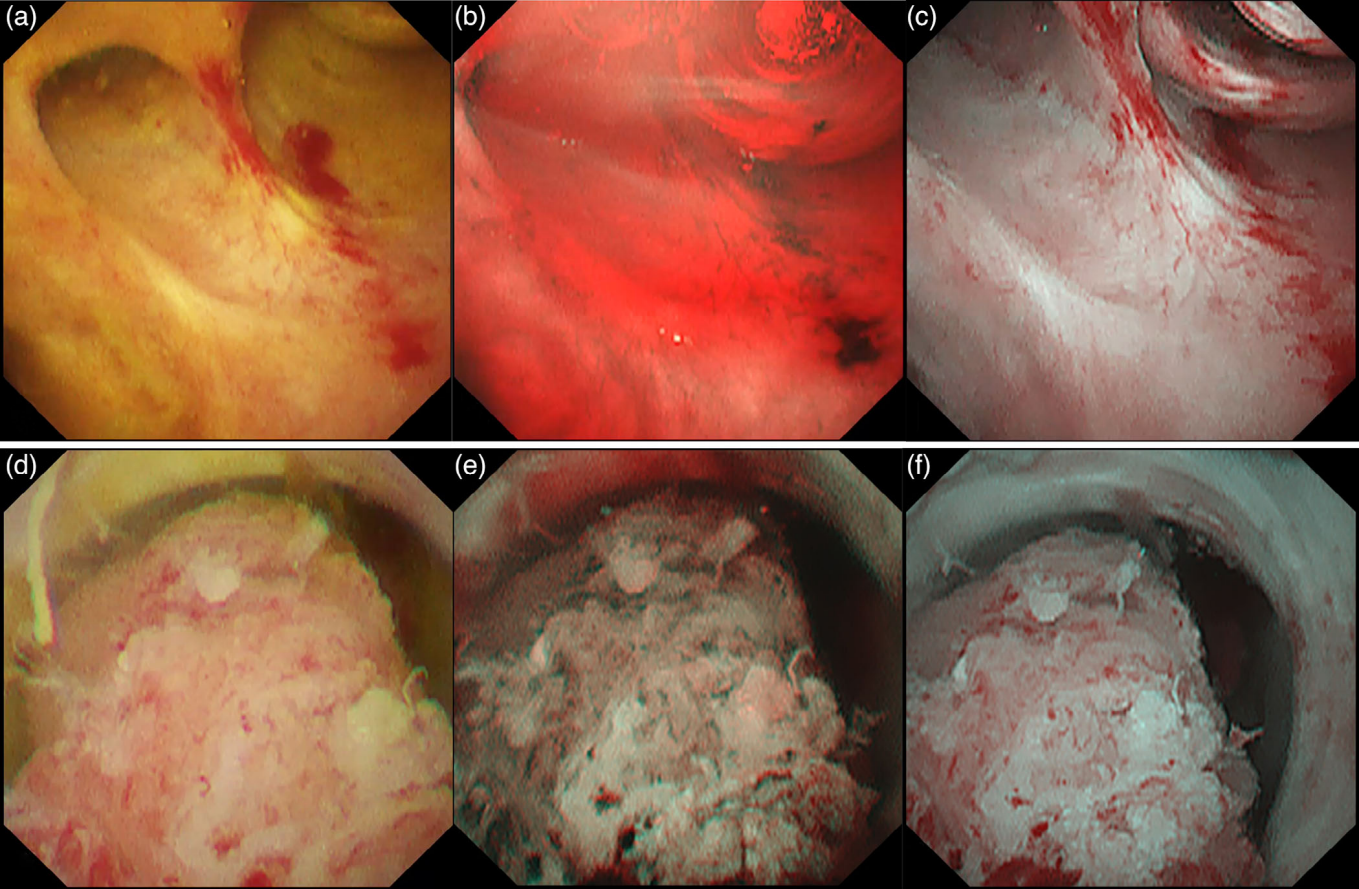
Red dichromatic imaging during peroral cholangioscopy. (a) White light imaging, (b) narrow‐band imaging: Bile is depicted in red color, significantly impairing the field of vision. (c) Red dichromatic imaging: Even in the presence of bile, it is possible to clearly visualize the bile duct walls and vessels. (d) Intraductal papillary neoplasm of the bile duct in white light imaging, (e) narrow‐band imaging, and (f) red dichromatic imaging Mode 3.

The cholangioscopy systems available for RDI are a combination of the latest scope, CHF‐B290 (Olympus Co.), and the latest endoscope system, EVIS X1 (Figure 10a–f).

Kimura et al.^28^ reported that RDI Mode 2 was effective in identifying the bleeding point of biliary hemorrhage, as observed in POCS.

RDI Mode 3 depicts deep thick vessels in green and superficial thin vessels in brown, resulting in images similar to those of NBI, allowing for clearer recognition of the vascular structures. A significant difference from NBI is that the bile is translucent in RDI Mode 3, which is considered superior for observing intraductal biliary lesions. There are several case reports on the usefulness of RDI Mode 3 for the observation of biliary lesions, with Koiwai et al.^29^ revealing the findings of lateral spread of IPBN, and Tanisaka et al.^30^ and Matsumoto et al.^31^ describing the identification of bile duct cancers, despite the presence of bile. However, there are only a small number of reports available, and no studies have compared RDI with other IEEs. Thus, further research is necessary.

### Digital method

The digital method involves image enhancement through signal processing and an image‐processing algorithm. Various algorithms have been proposed depending on the objectives and means of imaging, including one that enhances the contours or fine patterns in the constructed images, one that emphasizes image contrast, and one for color conversion. Digital methods typically include flexible spectral imaging color enhancement, i‐scan digital contrast, index of hemoglobin color enhancement, and TXI. Although TXI is a new technology, reports on its usefulness are beginning to increase not only in the GI field but also in the PB field.

#### Texture and color enhancement imaging

**FIGURE 11:**
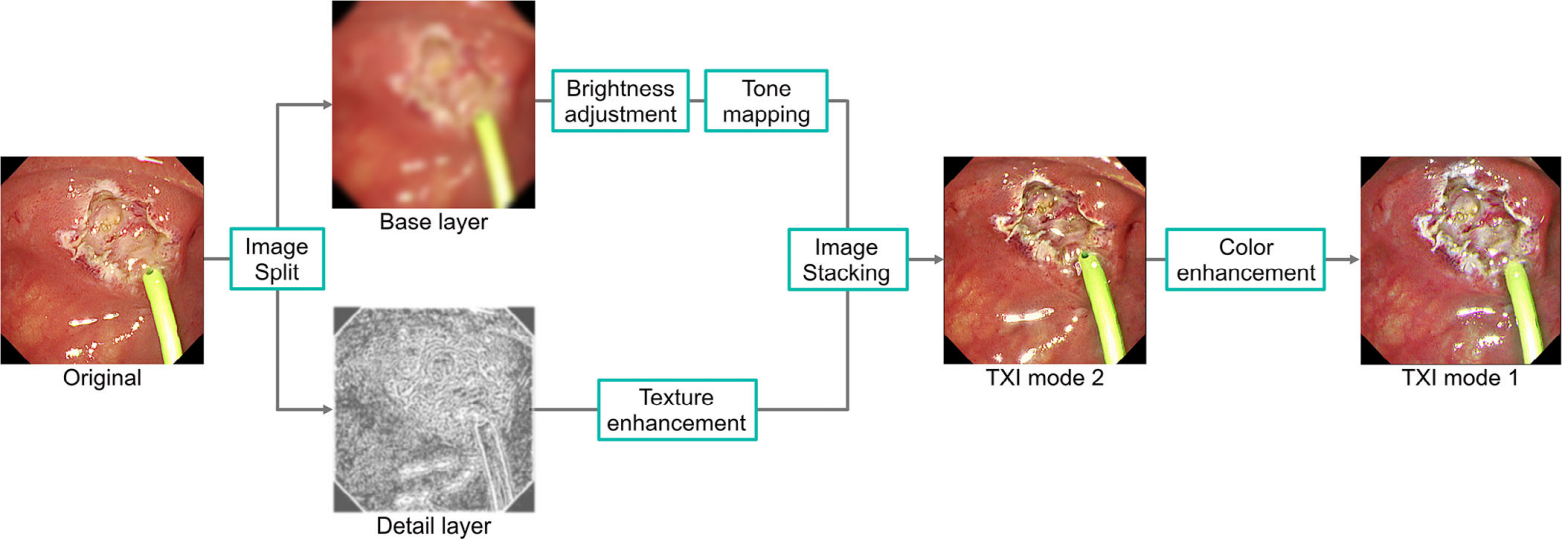
Main mechanism of texture and color enhancement imaging (TXI).

TXI was launched as a novel digital method IEE, equipped with a new endoscopic system. TXI was designed to enhance three image factors in WLI– texture, brightness, and color– to define subtle tissue differences clearly.^32^ Traditional image enhancement methods can improve the contrast of images; however, excessive enhancement may compromise the natural appearance of the image, resulting in the loss of details in both bright and dark areas, and may also accentuate noise. To avoid these issues, a TXI algorithm was developed by applying the principles of the Retinex theory.^33^ In the TXI algorithm (Figure 11), the original input image is split into two layers: a base layer and a detail (texture) layer. The brightness of the base layer is corrected and enhanced, and the texture of the detail layer is enhanced. The two enhanced layers are recombined and output as TXI Mode 2. TXI Mode 1 is the output after adding color‐tone enhancement to TXI Mode 2. The usefulness of the image enhancement features of TXI has been reported in various diagnostic and therapeutic situations in GI endoscopic practice. Recently, there has been an increasing number of reports on the effectiveness of TXI in the PB field.

##### Observation of PB orifices

**FIGURE 12:**
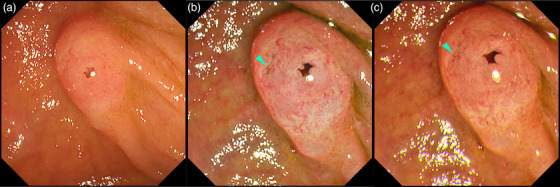
Texture and color enhancement imaging (TXI) for identifying the biliary orifice (green arrowhead) of a naïve papilla. (a) White light imaging, (b) texture and color enhancement imaging Mode 1, and (c) texture and color enhancement imaging Mode 2.

Accurate recognition of the structure of the duodenal papilla is essential for biliary/pancreatic cannulation, and TXI may contribute to improving the success rate of cannulation by enhancing the structure, brightness, and color tone, thereby enabling more accurate recognition of the orifice structure (Figure 12a–c).

Miyaguchi et al.^34^ reported that TXI Mode 1 could improve papillary recognition by trainees inexperienced in ERCP. With respect to distinguishing the separate and septal types of the papilla, TXI Mode1 had a higher concordance rate between trainees and experts than WLI. The enhancement of the structure in TXI makes it easier for even inexperienced trainees to distinguish between the separate and septal types, allowing for the formulation of appropriate cannulation strategies. This can lead to an improvement in the rate of intubation and a reduction in the risk of post‐ERCP pancreatitis; further research on this topic is anticipated in the future.

Tanisaka et al.^35^ conducted a multicenter retrospective cohort study to compare TXI and WLI during biliary cannulation in patients who underwent Roux‐en‐Y gastrectomy via short‐type single‐balloon enteroscopy‐assisted ERCP (SBE‐ERCP). The median time to successful biliary cannulation was significantly shorter with TXI than with WLI (*p* = 0.04). Although there was a tendency toward a higher cannulation success rate with TXI, the difference was not significant (TXI: 93.9% vs. WLI: 83.7%, *p* = 0.14).

**FIGURE 13:**
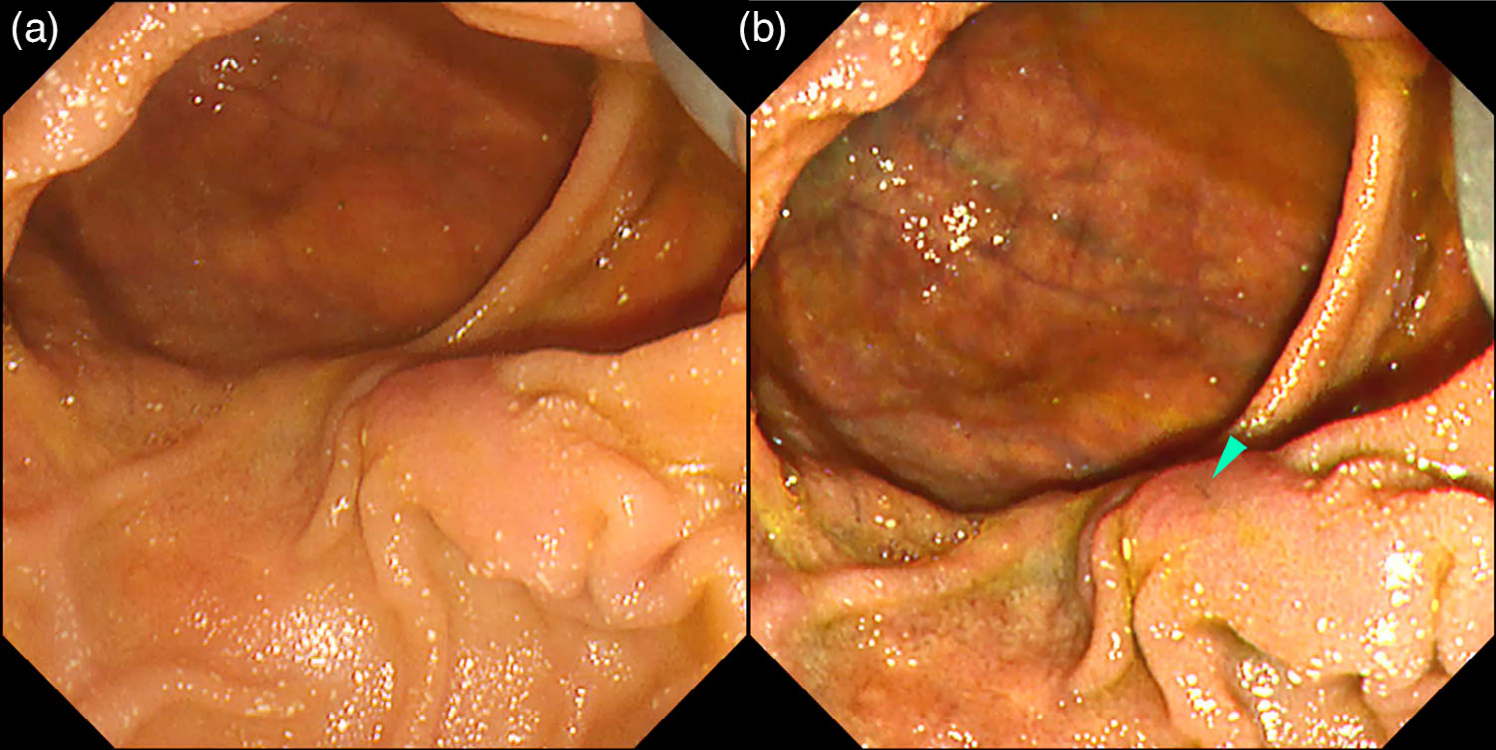
Texture and color enhancement imaging for detecting the biliary orifice (green arrowhead) of an interdiverticular papilla. (a) White light imaging and (b) texture and color anhancement imaging Mode 2. [Reprinted with permission from Georg Thieme Verlag KG, originally published in *Endoscopy* 2022^35^]

**FIGURE 14:**
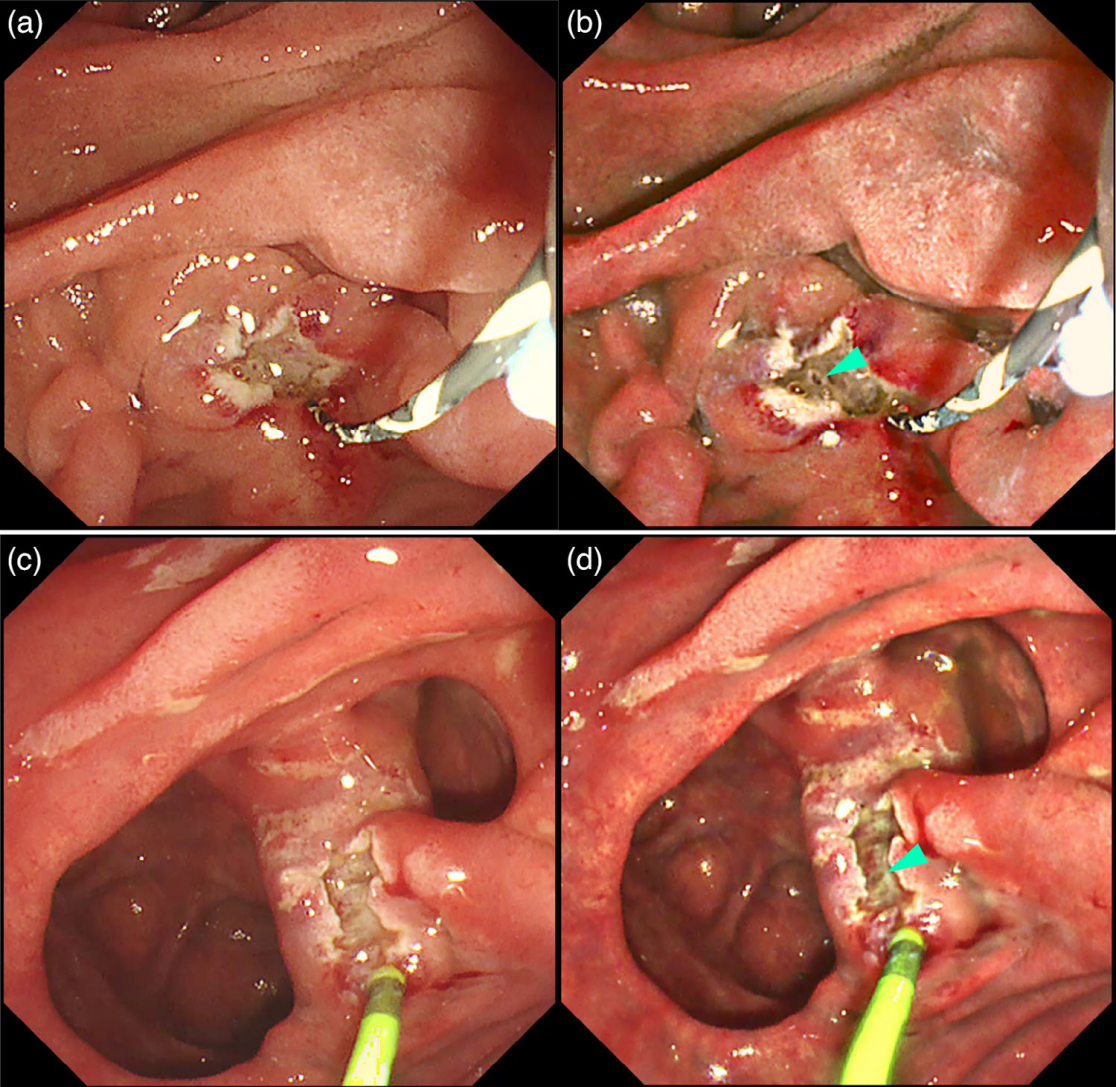
Texture and color enhancement imaging for detecting the biliary orifice (green arrowhead) after precut papillotomy. (a) White light imaging, (b) texture and color anhancement imaging Mode 2, (c) white light imaging, and (d) texture and color enhancement imaging Mode 2. [Reprinted with permission from Georg Thieme Verlag KG, originally published in *Endoscopy* 2022^36^]

TXI has been reported to be potentially useful in difficult situations, such as diverticular papillae, where the orifice is difficult to identify^36^, ^37^ (Figure 13a,b); orifices on the incision surface after a precut papillotomy in cases of difficult bile duct cannulation^15^, ^38^ (Figure 14a–d); or after endoscopic papillectomy.^39^, ^40^

Toyonaga et al.^38^ published a retrospective observational study that demonstrated the effectiveness of TXI Mode 2 in identifying the biliary orifice after needle knife precut papillotomy. The accuracy rate of initial identification of the biliary orifice was higher with TXI than with WLI (50.6% vs. 35.6%, odds ratio 2.26, *p* = 0.003). Compared with WLI, TXI had a higher visibility score (*p* < 0.001), and the color difference between the biliary orifice and surrounding tissue was more pronounced with TXI than with WLI (*p* < 0.001).

##### Ampullary tumors

**FIGURE 15:**
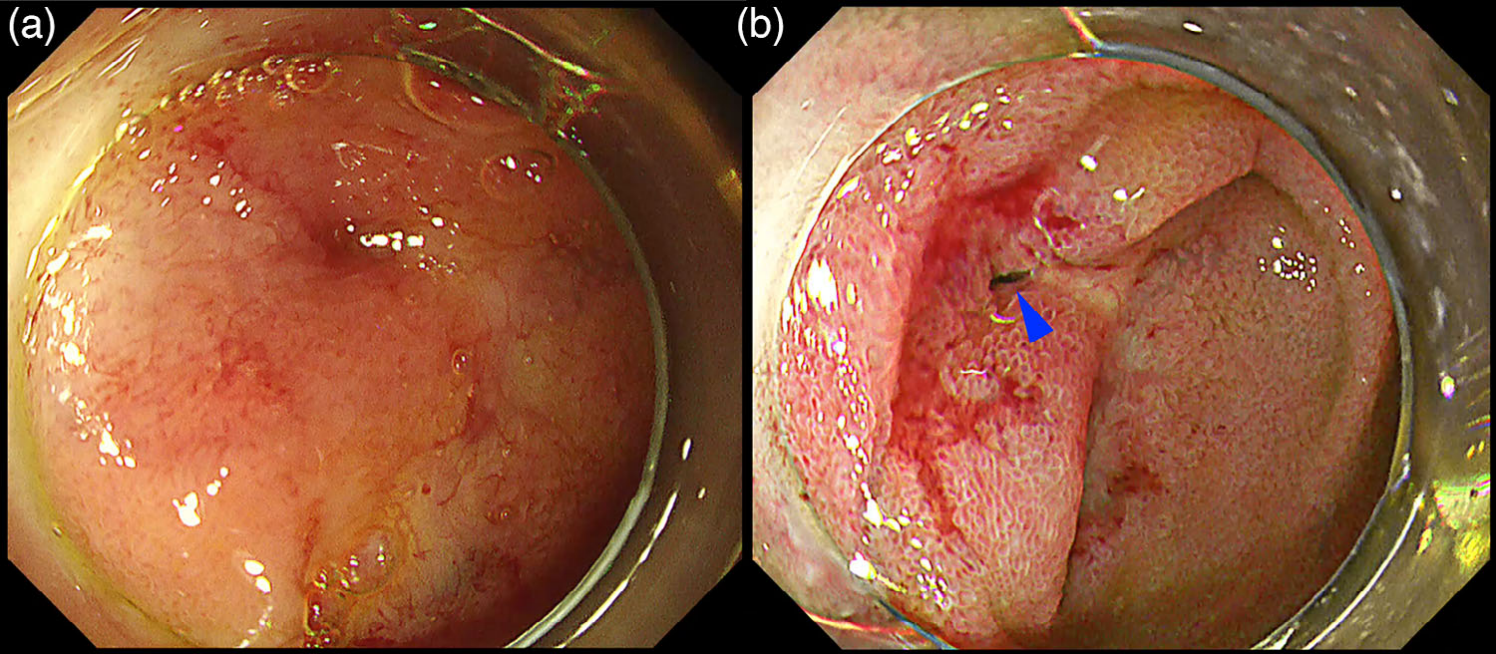
Texture and color enhancement imaging for recognition of boundaries of an ampullary tumor. (a) White light imaging, (b) texture and color enhancement imaging Mode 1, and (c) texture and color enhancement imaging Mode 2. [Reprinted with permission from John Wiley and Sons, originally published in *Dig Endosc* 2023^40^]

**FIGURE 16:**
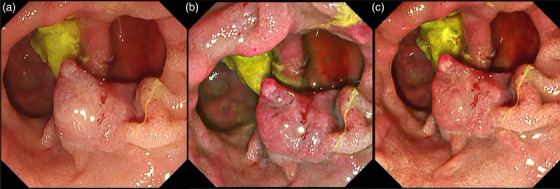
Texture and color enhancement imaging for recognition of the biliary orifice (green arrowhead) of an ampullary tumor. (a) White light imaging, (b) texture and color enhancement imaging Mode 1, and (c) texture and color enhancement imaging Mode 2.

**FIGURE 17:**
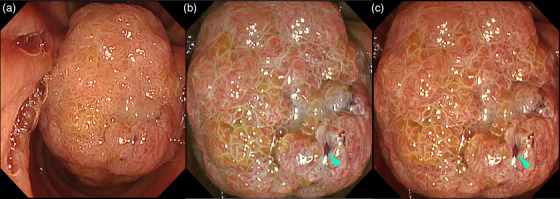
Texture and color enhancement imaging for detecting the biliary (green arrowhead) and pancreatic (blue arrowhead) orifice after endoscopic papillectomy, (a) White light imaging, (b) texture and color enhancement imaging Mode 2, (c) white light imaging, and (d) texture and color enhancement imaging Mode 2. [Reprinted with permission from John Wiley and Sons, originally published in *Dig Endosc* 2023^40^]

During the evaluation of lateral extension and endoscopic resection of papillary tumors, IEEs such as indigo carmine spraying and NBI are useful for making the boundaries easier to recognize. Toyonaga et al.^40^ claimed that TXI was also beneficial for recognizing boundaries (Figure 15a–c). Unlike indigo carmine, TXI does not require dye dispersion, and its color tone is not as distinct from that of WLI as NBI, which could be considered an advantage of TXI. Emphasizing the indentation of the orifice and the color of the bile could also contribute to the identification of the biliary orifice in ampullary tumors (Figure 16a–c). After papillectomy for ampullary tumors, it is necessary to ensure cannulation of the pancreatic or bile ducts to prevent complications. Taking time during the procedure can lead to loss of visibility due to bleeding from the incision edge, and applying pressure to the incision site may cause duodenal perforation. From this perspective, TXI is also useful for identifying the biliary and pancreatic duct orifices on the incision surface^39^, ^40^ (Figure 17a–d).

##### Detection of cholangio‐/pancreato‐jejunal anastomosis

It is difficult to identify the cholangio‐jejunal anastomotic site during BE‐ERCP in patients with postoperatively reconstructed intestines. Although indigo carmine spray can be effective, once applied, it cannot be immediately reversed and may instead reduce visibility. TXI, which emphasizes structure, brightness, and color tone, can improve the visibility of anastomotic sites that are difficult to identify.

**FIGURE 18:**
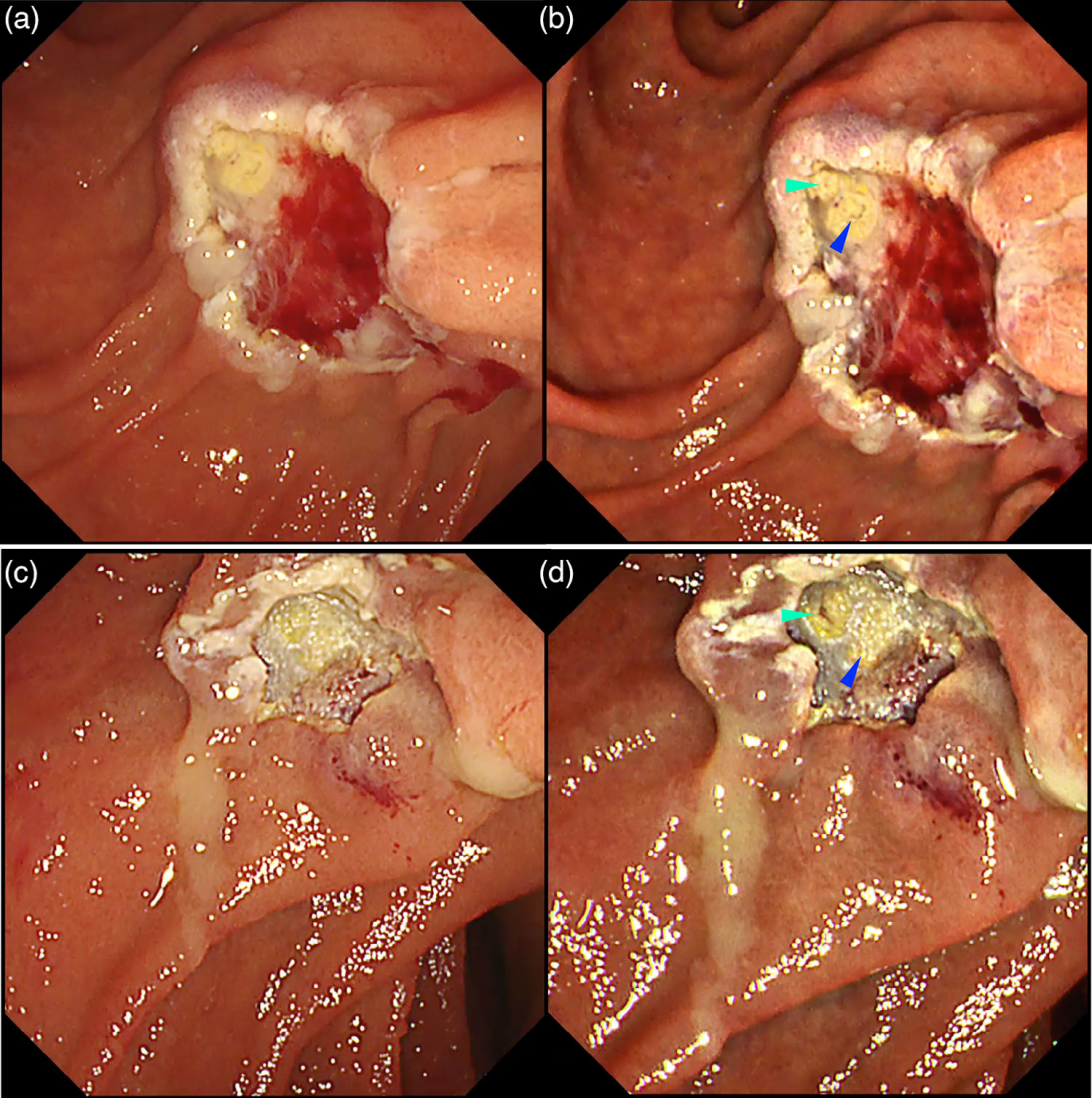
Texture and color enhancement imaging for identifying the pancreato‐jejunal anastomosis site (blue arrowhead). (a) White light imaging and (b) texture and color imaging Mode 1. [Reprinted with permission from Georg Thieme Verlag KG, originally published in *Endoscopy* 2022^41^]

TXI can be used in combination with a therapeutic short‐type single‐balloon enteroscope, such as the SIF‐H290S (Olympus Co.) and EVIS X1. Toyonaga et al.,^41^ Tanisaka et al.,^42^ and Takenaka et al.^43^ have reported that TXI is useful in identifying difficult cholangio‐jejunal anastomotic stenoses. The enhancement of shadows, color contrast, and structural alterations in the concavity of the anastomosis and surrounding areas may lead to improved visibility (Figure 18a,b). With the ability to switch between WLI and TXI by pushing a button, TXI can be considered an option when the anastomotic site is difficult to identify. However, no large‐scale studies have compared the anastomosis identification rates between WLI and TXI, and further research is warranted.

##### Peroral cholangioscopy

**FIGURE 19:**
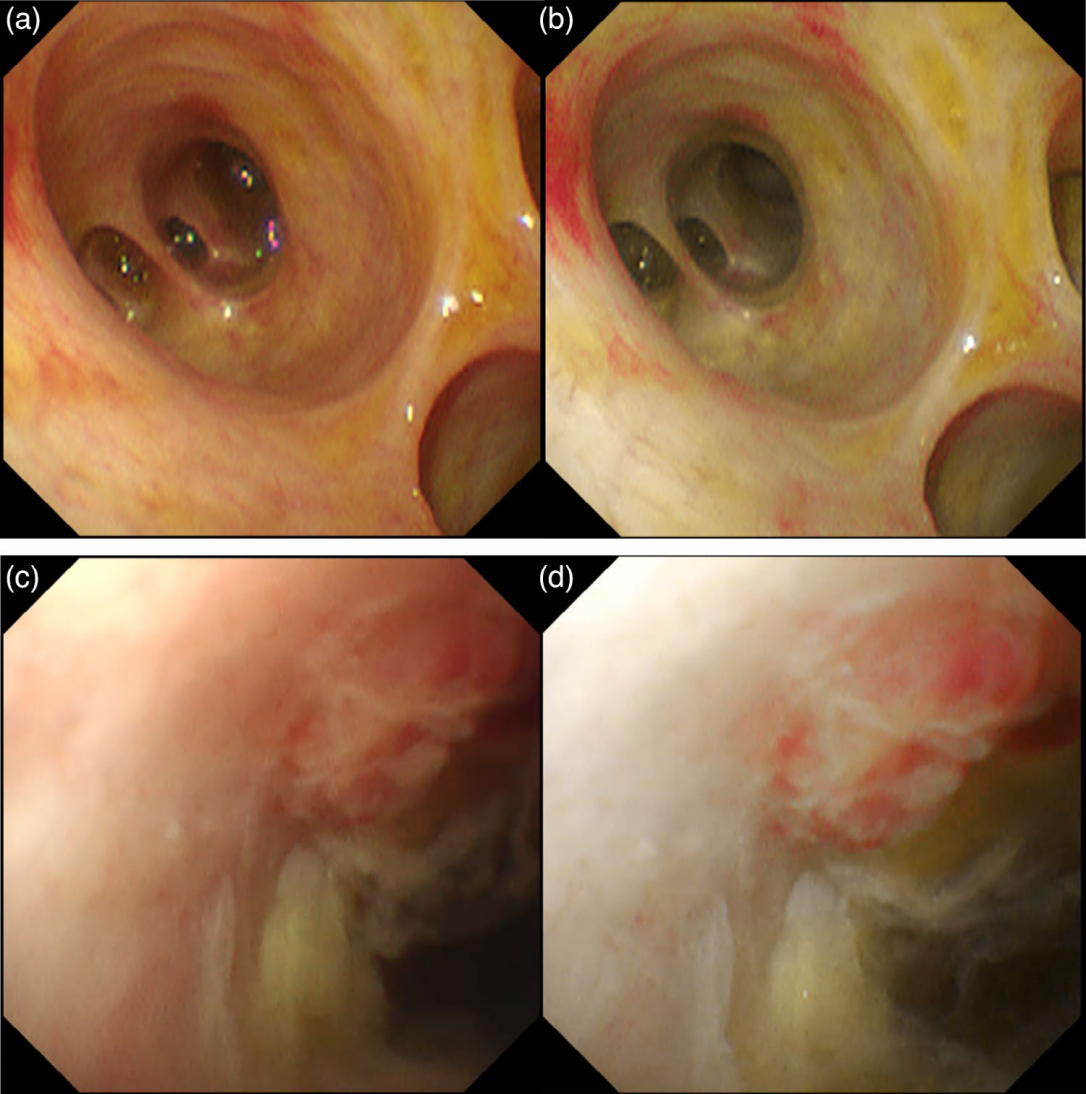
Texture and color enhancement imaging during peroral cholangioscopy. Enhanced color and brightness make it easier to recognize red areas or dilated vessels and detect lesions. (a) White light imaging, (b) texture and color enhancement imaging, (c) white light imaging, and (d) texture and color enhancement imaging.

TXI is also available in POCS in combination with CHF‐B290 and EVIS X1. Tanisaka et al.^30^ reported the usefulness of TXI during POCS for examining biliary strictures. The enhancement of structure, color tone, and brightness by TXI can also be useful in cholangioscopy for the recognition of boundaries between normal and diseased areas. Additionally, TXI is beneficial for the detection of skip lesions, which are prone to be overlooked (Figure 19a–d).

#### Virtual indigo carmine chromoendoscopy

In recent years, a new virtual chromoendoscopy technique based on artificial intelligence (AI) technology, known as the “Virtual indigo carmine chromoendoscopy” (VIC), has emerged. VIC uses a to highlight specific features in endoscopic images, thereby digitally replicating the dyeing effect of indigo carmine without the physical use of the dye.^44^ VIC aims to leverage the visibility advantages of indigo carmine staining while eliminating the hassles and risks associated with the use of dyes.

Indigo carmine staining is expected to be useful for diagnosing biliary diseases in POCS. However, during POCS, the bile duct must be perfused with saline solution, which means that indigo carmine spraying can obstruct the visual field by mixing with the perfusion fluid, and the safety of dispersing it into the bile duct has not been clarified.

Sato et al.^45^ reported the effectiveness of artificial intelligence‐mediated VIC in POCS of bile duct lesions. VIC helped to identify the margins and surface irregularities of malignant lesions in the bile duct, aiding in the accurate determination of the extent of resection required. This approach facilitates precise surgical intervention, leading to successful R0 resection of malignant lesions.

## CONCLUSIONS

This review summarizes the latest advancements in IEE in the PB field. Specifically, we discussed how new technologies, such as TXI and RDI, could improve the accuracy of endoscopic observation in the diagnosis and treatment of PB diseases. These technologies offer considerable advantages over traditional WLI, particularly in identifying difficult‐to‐detect lesions, biliary orifices, and bleeding points. We anticipate the further development of innovative IEE techniques for standard clinical use, potentially opening new frontiers in the management of PB diseases. However, reports on IEE in the PB field are mainly case reports and there is a lack of evidence; thus, further accumulation of cases and research is desirable.

## CONFLICT OF INTEREST STATEMENT

Akio Katanuma received honoraria for lectures from Olympus Co. (Tokyo, Japan). The other authors declare no conflict of interest.

## ETHICS STATEMENT

Not applicable.
